# LightSNiP assay is a good strategy for pharmacogenetics test

**DOI:** 10.3389/fphar.2015.00114

**Published:** 2015-06-02

**Authors:** Stefania Cheli, Filippo Pietrantonio, Emilio Clementi, Felicia S. Falvella

**Affiliations:** ^1^Unit of Clinical Pharmacology, Department of Biomedical and Clinical Sciences L. Sacco, “Luigi Sacco” University Hospital, Università di MilanoMilan, Italy; ^2^Medical Oncology Department, Fondazione IRCCS Istituto Nazionale dei TumoriMilan, Italy; ^3^Scientific Institute IRCCS Eugenio MedeaLecco, Italy; ^4^Unit of Clinical Pharmacology, Department of Biomedical and Clinical Sciences L. Sacco, CNR Institute of Neuroscience, “Luigi Sacco” University Hospital, Università di MilanoMilan, Italy

**Keywords:** SNP, simpleprobe, real-time PCR, melting, pharmacogenetics

The individual response to standard doses of drugs has a large variability depending on intrinsic factors (age, sex, and disease states) and/or extrinsic factors (diet, chemical exposures from the environment). The influence of these factors on drug responses has therefore to be taken into consideration when making decisions on treatment regimes (Thummel and Lin, 2014). Nonetheless, the taking into account of these factors limits, but does not eliminate, the high degree of interindividual variability in terms of efficacy or fatal adverse reactions. The individual genetic background, explains part of the different pharmacokinetic and pharmacodynamic drug responses both in term of efficacy and toxicity (Hertz and McLeod, 2013). Evidence indicates that genetic variations account for an estimated 20–40% of inter-individual differences in drug metabolism and response (Karczewski et al., 2012). The commonest genetic variations are single nucleotide polymorphisms (SNPs), representing approximately 90% of all human genetic variations and occurring every 100–300 base pairs (Crews et al., 2012). Some of these polymorphisms have been identified for many proteins including enzymes, drug receptors, transporters, and targets of the commonest drugs. These polymorphisms can cause alterations in the amount, structure, binding, and/or function of these proteins, influencing how drugs interact with them (Ma et al., 2012; Patel et al., 2013). The study of these genetic variants and their role in improving drug efficacy and reducing side effects, termed pharmacogenetics, is now established in the clinical practice for many drugs including abacavir, irinotecan, and 6 mercaptopurines (Ingelman-Sundberg, 2008; Wang et al., 2011). To facilitate an appropriate clinical implementation of pharmacogenetics, guidelines have been published by the Clinical Pharmacogenetics Implementation Consortium (CPIC) and the Dutch Pharmacogenetics Working Group (DPWG). The aim of these guidelines as they clearly state is to facilitate translation of the genetic laboratory test results into prescribing decisions for specific drugs, or class of drugs, such as vitamin K antagonist, tricyclic antidepressants (Johnson et al., 2011; Caudle et al., 2013; Hicks et al., 2013). So far, several techniques have been described to detect specific SNPs, with the Sanger Sequencing, the Denaturing Gradient Gel Electrophoresis (DGGE), the Single Strand Conformational Polymorphism analysis (SSCP), the Pyrosequencing and Sequenom being the most widely employed. Recently also techniques of high-throughput screening and for large-scale characterization of SNPs have been developed; these platforms, however, are expensive, not flexible and not of practical use for small to medium size laboratories. More user-friendly, SNPs detection methods for pharmacogenetic tests are based on PCR amplification, in conjunction with an appropriate probe technology (real-time PCR) as TaqMan, Scorpion and SimpleProbe®. Among these, SimpleProbe® appears of particular interest. SimpleProbe format is composed of one hybridization probe, labeled with a fluorophore; this oligonucleotide is designed spanning the variant of interest, but does not participate into the amplification process. Once hybridized to its target sequence, the SimpleProbe probe releases more fluorescence than when not hybridized. Changes in fluorescence that are exclusively based on the probe hybridization status are detected by melting curve analyses. Any mismatch positioned under the SimpleProbe® will reduce the hydrogen-bonds, hence the melting temperature, thus enabling analysis of polymorphisms. Diverse variations destabilize the hydrogen-bonds differently, usually yielding different and specific melting points, thus making it possible to identify polymorphisms tightly close to the intended variant, giving it potential advantages over competing technologies.

Here, we describe the advantages of real-time PCR assays for the detection of three SNPs selected for their involvement in DPD and UGT1A1 enzymes regulations (Falvella et al., 2015). This SNP detection is based on LightCycler Technology with SimpleProbe® probes (LightSNiP assays); it designed by TIB Molbiol (Berlin, Germany) and is a validated Real Time PCR method used in many areas of molecular diagnostics. From amplification and detection with specific probes by melting curve analysis, it is possible to obtain a visual discrimination of normal and variant alleles in the homozygous and heterozygous status. When selecting the most appropriate technique for a analysis of a given genotype, few general considerations should be taken into account such as correct detection, simplicity in the technology and reproducibility.

## Example 1. rs8175347 (UGT1A1^*^28)

**Figure 1:**
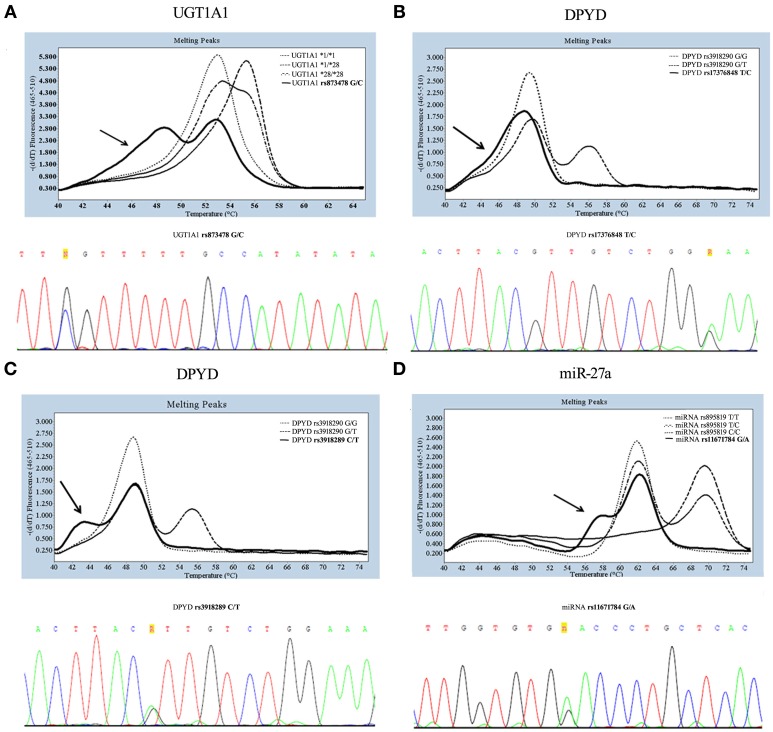
**Genotyping and sequencing of three SNPs: UGT1A1 sequence around c.-64G>C (A), DPYD sequence around rs3918290 (B,C), miR-27a rs895819 (D)**. In the melting curve analysis obtained by LightSNiP probe during genotyping, the common genotype (wild-type) are indicated by dot and heterozygous by dash-dot style, while the arrows indicate samples of rare genotype. In the panel of direct sequencing, letters **“R”** and **“n”** indicate additional polymorphisms.

UGTs are important determinants of drug responses influencing glucuronidation (Rowland et al., 2013); the best known of them is the *UGT1A1* gene. A common promoter (TA)n polymorphism (UGT1A1^*^28, *7TA* repeats) significantly decreases *UGT1A1* gene transcription, leading to reduced glucuronidation, in turn increasing toxicity of SN-38, the active metabolite of irinotecan (Innocenti et al., 2014). Because of this, UGT1A1^*^28 has now become a biomarker of neutropaenia, and its analysis is inserted into the irinotecan package. Two others alleles in the *UGT1A1* promoter region, UGT1A1^*^36 (TA)5 and UGT1A1^*^37 (TA)8, have been associated to altered enzyme function and may be detected by the hybridization probes (von Ahsen et al., 2000); however their occur almost exclusively in populations of African origin (Beutler et al., 1998). In addition, genotyping of UGT1A1^*^28 by LightSNiP can also identify a very rare allele positioned in close proximity. As shown in Figure 1A (upper panel), the sample indicated by the arrow presented a different temperature profile in one of the two peaks obtained: while the melting peak of 52.97°C corresponded to the UGT1A1^*^1 common allele (6TA repeats) we found one peak at 48.09°C, different from the expected peak at 53.72°C for UGT1A1^*^28, unmasking the existence of another polymorphism. By direct sequencing, we were able to identify the presence of the *UGT1A1* “*C*” allele in heterozygosis in c.-64 position (rs873478 G>C) (Figure 1A, lower panel).

## Example 2. rs3918290 (DPYD^*^2A)

Genetic variations in *DPYD* have been identified as a major contributor to Dihydropyrimidine dehydrogenase (DPD) deficiency. The most studied *DPYD* variation, catalytically inactive, is the ^*^2A (rs3918290) leading to reduced clearance of 5-fluorouracil (5-FU) and increased frequency of severe toxicity (Johnson et al., 2002). Genotyping of the DPYD^*^2A variant by LightSNiP assay may be accompanied by unusual types of curves in the correspondence of the expected peak of the common allele (49.37°C). In particular, melting curve analyses displayed either a “baggy” (Figure 1B, upper panel) or a “bulge” (Figure 1C, upper panel) curve. These melting profiles suggest that one or more bases remain unpaired. The sequencing analysis confirmed the presence of an as yet undetected polymorphisms. Particularly, the “baggy” curve represents the *DPYD* c.1896 (rs17376848) variant allele (Figure 1B, lower panel) and the “bulge” curve the *DPYD* c.1905 (rs3918289) variant allele (Figure 1C, lower panel), both in heterozygosis.

## Example 3. rs895819 (miR-27a)

DPD expression may be regulated at the post-transcriptional level. Recent data have suggested that mapping of rs895819 in miR-27a may be useful to determine 5-FU sensitivity, since it has been associated with a reduced DPD enzyme activity (Offer et al., 2014). As expected, genotyping of rs895819 showed a melting peak at 62.98°C for the common allele and at 70.34°C for the variant allele. As shown in Figure 1D (upper panel), the sample indicated by an arrow in correspondence to the expected peak at 62.98°C presented a “bulge” melting curve. Also in this case the sequencing analysis revealed the presence in heterozygosis of rs11671784, another polymorphism tightly close to the one in study (Figure 1D, lower panel).

## Clinical utility

The predictive power of pharmacogenetic tests depends on the contribution of the genetic polymorphisms on the expression and/or function of the gene involved in the pharmacological response, the frequency of the variants tested and the presence of alternative pharmacokinetic pathway(s). To date, some anticancer drugs approved by the US FDA, including irinotecan and 5-fluorouracil, require a germline pharmacogenetic testing prior to their administration, as indicated in their prescription drug labeling. This report highlights that how pharmacogenetic testing is carried out is critical since the choice of a specific method may be instrumental in unmasking factors that influence the response to these drugs and that go unnoticed with other methods.

Concerning irinotecan, polymorphisms within *UGT1A1* have been shown to reduce glucuronidation of the active metabolite SN-38, thus leading to an increase in its circulating levels and an increased risk of severe neutropaenia; the UGT1A1^*^28 genotype can thus be used to individualize dosing of irinotecan (Innocenti et al., 2014). Since polymorphisms in the promoter region of the coding gene have been reported associated to transcriptional regulation, a genotyping method able to detect the existence of SNPs close to the promoter functional region is of choice. The rs873478 (c.-64G>C) polymorphism is close to the UGT1A1^*^28 allele; its allele frequencies is 1% globally, reaching as high as 4% in East Asian Individuals (1000 genomes). The role in gene expression of rs873478, located 11 base pairs upstream the TATA box, is still not known (Yea et al., 2008); thus, methods that detect it alongside the other UGT1A1^*^28 polymorphism are useful tools to establish its clinical relevance.

Genetic variations in the DPD gene (*DPYD*), coding for a key enzyme involved in the catabolic pathways of fluoropyrimidines, have been recognized as major contributors to enzyme deficiency and fluoropyrimidines-associated toxicity. Some polymorphisms in *DPYD* gene have been reported in association with reduced enzyme activity. In particular, the genotyping of c.1905 + 1G > A (^*^2A), c.1679T > G, and c.2846A > T is recommended in the clinical practice to prevent severe toxicity; they however explain only a small percentage of the toxicity due to low allelic frequency (Johnson et al., 2002; Morel et al., 2006). Other *DPYD* variants linked to fluoropyrimidines toxicity have been described, including the *DPYD* c.1896 variant close to *DPYD* ^*^2A (Teh et al., 2013). The frequency of this variant is high, reaching 6% in the global population, and its detection has therefore acquired clinical significance. The SimpleProbe® technology allows the identification in DPYD^*^2A of the c.1896 variant alongside the other ones. A new genotyping assay, developed specifically for a *DPYD* c.1896 confirmed what was seen with sequencing and “baggy” curve.

Intra-individual difference in DPD expression may be due also to epigenetic factors. miR-27a and miR-27b repress DPD expression and therefore are important in cellular sensitivity to 5-fluorouracil (Offer et al., 2014); rs895819 maps in the coding region of the hsa-mir-27a hairpin and is quite common being present in up to 35% of the global population (1000 genomes). The variant allele at rs895819 results in a loop region larger than the common hairpin and therefore more effectively processed, thus enhancing mature miR-27a expression (Offer et al., 2014). When we genotyped rs895819 by LightSNiP we were able to detect also rs11671784, another polymorphism very close to rs895819. The miR-27a rs11671784 polymorphism is located at a distance of only 4 nucleotides and represents a rare variant, consisting of a G >A nucleotide substitution that results in a change from a G:C complementary pair to a G:U mismatch in the stem region of miR-27a precursor (Yang et al., 2014). This variant has been reported to impair the maturation of miR-27a, resulting in a reduced expression of mature miR-27a and reduced gastric cancer risk (Yang et al., 2014). A genotyping assay able to detect at the same time rs895819 and the rare variant rs11671784 is thus a real clinical need. In this respect it is important to emphasize that the TaqMan allelic discrimination assay does not detect this variant (Yang and Burwinkel, 2012).

The three examples described highlight also some methodological aspects relevant in the clinical perspective. The “ideal” genotyping method needs to meet several quality specifications. In particular, the method has to be easily and quickly developable by the DNA sequence, with a low cost of assay development for both reagents and time spent for optimization, and make use of a reaction capable of amplifying correctly also non-optimal DNA samples. It also has to be automated and with minimal hands-on operation, with simple data analysis and a flexible and scalable reaction format (Kwok, 2001).

Here we show that, at least for the specific genes and polymorphisms we analyzed, LightSNiPs probes are a highly sensitive and rapid tool for SNP genotyping because they readily identify both frequent and rare variant alleles with a single short probe. Additionally, the LightSNiP assay detects other polymorphisms under the hybridization probe and in the proximity of the intended SNP, through the generation of abnormal or unexpected melting curve point. Once these are characterized, as we did, by sequencing analysis their interpretation in subsequent tests becomes immediate and thus of diagnostic usefulness. We do not imply to establish a superiority of LightSNiP vs. other methods, for which, we would have had to carry out a full comparison analysis; we just highlight a good intrinsic quality of the genotyping-method significant for routine diagnostic pharmacogenetics activity. Based on the features and the ease of use of SimpleProbe® technology, we suggest that the LightSNiP assay is a good strategy for pharmacogenetics analysis to provide the clinician a correct results in a short time.

### Conflict of interest statement

The authors declare that the research was conducted in the absence of any commercial or financial relationships that could be construed as a potential conflict of interest.
